# Proton Pump Inhibitors Display Antitumor Effects in Barrett's Adenocarcinoma Cells

**DOI:** 10.3389/fphar.2016.00452

**Published:** 2016-11-25

**Authors:** Eduardo Chueca, Nadezda Apostolova, Juan V. Esplugues, María A. García-González, Ángel Lanas, Elena Piazuelo

**Affiliations:** ^1^CIBERehdMadrid, Spain; ^2^Instituto de Investigación Sanitaria AragónZaragoza, Spain; ^3^Department of Pharmacology, University of ValenciaValencia, Spain; ^4^CIBA, Instituto Aragonés de Ciencias de la SaludZaragoza, Spain; ^5^Department of Medicine, Psychiatry and Dermatology, University of ZaragozaZaragoza, Spain

**Keywords:** Barrett's esophagus, esophageal adenocarcinoma, proton pump inhibitors, vacuolar ATPase, reactive oxygen species

## Abstract

Recent evidence has reported that proton pump inhibitors (PPIs) can exert antineoplastic effects through the disruption of pH homeostasis by inhibiting vacuolar ATPase (H^+^-VATPase), a proton pump overexpressed in several tumor cells, but this aspect has not been deeply investigated in EAC yet. In the present study, the expression of H^+^-VATPase was assessed through the metaplasia-dysplasia-adenocarcinoma sequence in Barrett's esophagus (BE) and the antineoplastic effects of PPIs and cellular mechanisms involved were evaluated *in vitro*. H^+^-VATPase expression was assessed by immunohistochemistry in paraffined-embedded samples or by immunofluorescence in cultured BE and EAC cell lines. Cells were treated with different concentrations of PPIs and parameters of citotoxicity, oxidative stress, and autophagy were evaluated. H^+^-VATPase expression was found in all biopsies and cell lines evaluated, showing differences in the location of the pump between the cell lines. Esomeprazole inhibited proliferation and cell invasion and induced apoptosis of EAC cells. Production of reactive oxygen species (ROS) seemed to be involved in the cytotoxic effects observed since the addition of N-acetylcysteine significantly reduced esomeprazole-induced apoptosis in EAC cells. Esomeprazole also reduced intracellular pH of tumor cells, whereas only disturbed the mitochondrial membrane potential in OE33 cells. Esomeprazole induced autophagy in both EAC cells, but also triggered a blockade in autophagic flux in the metastatic cell line. These data provide *in vitro* evidence supporting the potential use of PPIs as novel antineoplastic drugs for EAC and also shed some light on the mechanisms that trigger PPIs cytotoxic effects, which differ upon the cell line evaluated.

## Introduction

Barrett's esophagus (BE; El-Serag et al., 2004) is the main risk factor known for developing esophageal adenocarcinoma (EAC; Tytgat, 1995). The sequence of the carcinogenetic process in BE is well-known, starting from non-dysplastic BE (ND) to low-grade dysplasia (LGD), high-grade dysplasia (HGD), and finally invasive EAC (Jankowski et al., 2000). Despite combined therapies, EAC has poor prognosis with a 5-year survival rate of <20% (Hur et al., 2013), which is why the research of novel therapeutic strategies remains mandatory.

Proton pump inhibitors (PPIs) are the most widely used drugs in the treatment of BE. Their effect is mediated by an inhibition of the gastric proton pump, increasing the pH in the stomach and clearly relieving reflux symptoms (Klinkenberg-Knol et al., 1994). However, growing evidence suggests that PPIs, besides their role in reducing gastric acid secretion, may also act as antineoplastic agents targeting the excess acid production in cancer cells through the inhibition of vacuolar-ATPases (V-ATPases). Intracellular alkalinization associated with microenvironment acidification is an important hallmark of cancer cells and has been related to invasion, metastasis, proliferation, and resistance to chemotherapy (Raghunand et al., 2001; Luciani et al., 2004; Rofstad et al., 2006). The reversal of the aberrant pH gradient in cancer cells has been linked to decreased tumor growth and inhibition of spontaneous metastases (Robey et al., 2009).

V-ATPases seem to play a key role in homeostasis of tumor pH. Thus, increased levels of V-ATPase expression and plasma cell membrane location have been related to higher metastatic potential (Sennoune et al., 2004). In addition, several human tumors have shown to overexpress V-ATPases, especially those which display multirresistance to cytotoxic drugs (Chow and Hedley, 1997; Murakami et al., 2001; Luciani et al., 2004; Marino et al., 2010). For this reason, various studies have exposed the possibility of using V-ATPases as new targets in cancer treatment (Marquardt and Center, 1991; Martínez-Zaguilán et al., 1999; Sennoune et al., 2004; Yeo et al., 2004; Lu et al., 2005; De Milito et al., 2010).

Given that PPIs are drugs of choice in the treatment of acid-related diseases and have also shown antineoplastic effects through their ability to inhibit V-ATPases (Jankowski et al., 2000; El-Serag et al., 2004) we thought of great interest to elucidate the potential antineoplastic effects of PPIs on EAC cells.

We therefore conducted an *in vitro* study to assess whether the PPI esomeprazole is able to exert antineoplastic effects on three EAC cell lines, and also the cellular mechanisms involved in those effects. We evaluated the expression and subcellular location of V-ATPase in these cell lines, and the effects of different concentrations of esomeprazole on proliferation, apoptosis, intracellular pH (pHi), cell invasion, reactive oxygen species (ROS) production, and induction of autophagy.

## Materials and methods

### Drugs

Esomeprazole magnesium hydrate, omeprazole, N-acetylcysteine (NAC), thapsigargin (TG), RPMI-1640, MCDB-153 medium, and antibiotics were from Sigma-Aldrich (Madrid, Spain). Fetal bovine serum (FBS) and Hank's balanced salt solution (HBSS) were both from Life Technologies (Madrid, Spain). All compounds except pepstatin A, which was dissolved in 100% ethanol and NAC, which was dissolved in culture media, were dissolved in DMSO and made up with the media so that the final concentration of the vehicle was not >0.04% (v/v).

### Cell lines and culture conditions

Three EAC cell lines were used in this study. SK-GT-4 cell line (DMSZ, Braunschweig, Germany) was originally isolated from an adenocarcinoma of the distal esophagus. OE33 cell line (ECACC, Salisbury, UK), established from an adenocarcinoma of the lower esophagus arising in BE and OACM5.1C cells, established from a lymph node metastasis derived from a primary adenocarcinoma of distal esophagus with the presence of BE were both purchased from ECCAC (Salisbury, UK). EAC cells were cultured in RPMI-1640 supplemented with antibiotics (100 U/mL penicillin, 100 μg/mL streptomycin, and 0.25 μg/mL amphotericin B) and 10% FBS. A non-dysplastic BE derived cell line CP-A (ATCC, Teddington, USA) was used as a control to evaluate whether the effects of esomeprazole were specific of tumor cells. CP-A cells were cultured in MCDB-153 medium supplemented with 0.4 μg/L hydrocortisone (Sigma), 4 mM glutamine (ATCC), 20 mg/mL adenine (Sigma-Aldrich), 0.1 pM cholera toxin (Sigma-Aldrich), 5 μg/mL insulin, 5 μg/mL transferrin, 5 ng/mL selenium (Sigma), 150 μg/mL BPE (Sciencell), 20 ng/mL EGF (Sciencell), 100 U/mL penicillin, 100 μg/mL streptomycin, and 0.25 μg/mL amphotericin B, and 5% FBS, as previously described (Peréz-Sayáns et al., 2010).

### V-ATPase staining in the carcinogenic sequence of BE: immunohistochemistry

Immunohistochemistry was performed in 21 paraffin-embedded biopsies collected using strict endoscopic and histological criteria. Archival specimens were obtained from the Pathology department in Hospital Universitario Miguel Servet (Zaragoza). Samples were obtained from patients with BE showing different degrees of dysplasia, according to Riddell's classification criteria. Human duodenum samples were included as columnar epithelium controls.

2.5 μm tissue sections were cut, deparaffinized, rehydrated, and subjected to epitope retrieval using PT-Link module (Dako, Barcelona, Spain). The samples were then incubated with primary antibodies to V-ATPase subunit C1 (Santa Cruz Biotechnology, Dallas, USA) at 1/50 dilution using an automatic staining system (Dako Autostainer Plus) and counter-stained with hematoxylin and eosin. Slides were examined using the Envision Flex HRP system (Dako) and images were obtained using LAS EZ software (Leica, Barcelona, Spain) with a Leica DM 2500 microscope.

### V-ATPase expression in cell lines by confocal microscopy

To determine the subcellular location of V-ATPase, cells were double stained targeting both the pump and cell boundaries. CP-A, OE33, and SK-GT-4 cells were fixed in methanol, and OACM5.1C cells were fixed in 3% PFA. Cells were incubated with primary antibody (1:50 Goat polyclonal antibody against human V-ATPase subunit *a*, Santa Cruz) in 1% PBS-BSA followed by incubation with secondary antibody (1:500 Alexa fluor 488, Molecular Probes). Cells' boundaries were determined using antibodies against a pool of cytoqueratins (Dako) in CP-A and SK-GT-4 cells or E-Cadherin (Dako) in OE33 cells and subsequently incubated with secondary antibody (1:1000 Alexa Fluor 546, Molecular Probes). Alternatively, OE33 edges were labeled using Cell Mask plasma membrane stain (Life Technologies) and OACM5.1C limits were labeled with Phalloidin Alexa 546 (Molecular Probes).

Cells were mounted in Fluoromount G (Southern Biotech, Birmingham, USA) + Draq5 (Abcam, Cambridge, UK) and images were recorded with a confocal laser scanning microscope (Leica TCS SP2) with a 63x objective.

### Apoptosis assay

The effects of esomeprazole on apoptosis were assessed by flow cytometry using FACSAria cytometer (BD, Madrid, Spain). Cells were stained with Annexin V-FITC and propidium iodide (PI). Apoptotic cells were defined as Annexin V and Annexin V+PI positive cells. Cells were seeded in 25 cm^2^ cell culture flasks and cultured until reaching 40–50% confluence. Then, cells were treated with esomeprazole (0–200 μM under physiological (pH 7.4) or acidic (pH 6.5) conditions for 48 or 24 h, respectively, and collected for apoptosis determination. The experiments were repeated four times.

### Proliferation assay

Cell proliferation was measured using a BrdU assay kit (Roche, Barcelona, Spain) according to the manufacturer's manual. Briefly, BE and EAC cells were seeded in 96-well plates and the next day esomeprazole (0–200 μM) was added in physiological (pH 7.4) or acidified (pH 6.5) medium. After 48 or 24 h (physiological or acidified medium, respectively), cells were labeled with BrdU solution for 4 h and the labeling signal was quantified by measuring the relative absorbance (A_450_–A_690_ nm). Each assay was done in triplicate and the experiment was performed at least three times.

### Cell invasion assay

The effects of esomeprazole on OE33, SK-GT-4, and OACM5.1C invasiveness were tested using 100,000 cells per well by the quantitative CytoSelect^TM^ 96-well Cell Invasion Assay (Cell Biolabs, San Diego, USA) following the manufacturer's manual. In brief, cells were seeded in serum-free medium in the upper chamber of a modified Boyden chamber coated with a uniform layer of dried membrane matrix solution and allowed to invade toward 10% FBS for 24 h. Invasive cells that were able to degrade the matrix proteins in the layer, and ultimately pass through the pores on the bottom side of the membrane were stained and quantified as mean relative fluorescence units (RFUs) of repeated experiments (*n* = 7) measured at 480/520 nm using the Synergy HT plate reader (Biotek, Winooski, USA).

### Evaluation of cytosolic pH

pHi was evaluated in OE33, CP-A, and OACM5.1C cells by flow cytometry using the pH-sensitive fluorescent probe BCECF-AM (Invitrogen) as previously described (Chung et al., 2011). Cells were cultured with esomeprazole (0–200 μM) for 24 h. Then, cells (10^6^ cells/mL) were incubated with 2 μg/mL BCEFC AM, in PBS for 15 min. pHi was determined by the 525/640 nm fluorescent ratio with a FACSAria cytometer following the nigericin calibration procedure (Palanca-Wessels et al., 1998).

### Evaluation of ROS

The analysis of ROS production was assessed in OE33 and OACM5.1C cells at different time points after esomeprazole addition using a quantitative assay (Abcam, Cambridge, UK) based on ROS-sensitive probe DCFDA. Twenty-five thousand cells per well were seeded in 96-well plates and the next day, DCFDA probe and esomeprazole (0–200 μM) were added and incubated at 37°C. Intracellular ROS levels were evaluated every 60 min during 6 h and quantified as RFUs with respect to control cells measured at 495/529 nm using the Synergy HT plate reader (Biotek). In parallel, in order to evaluate if the addition of the antioxidant NAC was able to reduce ROS levels, we repeated the experiment pre-incubating cells with 5 mM NAC for 45 min before the addition of esomeprazole.

### Mitochondrial membrane potential (ΔΨ_m_)

OE33 and OACM5.1C cells were treated with esomeprazole (1–200 μM) or vehicle for 24 h. Treatment was removed and fluorochromes were added for further 30 min incubation (2.5 μM Hoechst and TMRM, both from Molecular Probes). Fluorescence was visualized using a fluorescence microscope (IX81, Olympus, Hamburg, Germany). “CellR” software was employed and the fluorescent signal was quantified using static cytometry software “ScanR” (Olympus). The fluorescence detection filter was excitation 540/10 nm, dichroic filter 570 nm and emission 590 nm.

### Cytochrome C quantification

We evaluated the release of cytochrome C to cytosol in the tumor cell line OE33 using the Cytochrome c Human ELISA Kit (Abcam) following the manufacturer instructions. Briefly, cells were treated with esomeprazole (5–200 μM) or the vehicle for 24 h. Cells and supernatants were collected, washed and lysed using a soft lysis buffer which dissolves plasma membrane without affecting intracellular membranes thus allowing the evaluation of the cytochrome C released from mitochondria. Absorbance was quantified with a plate reader (Synergy HT) at 450 nm, using 650 nm as a reference wavelength. Each sample was evaluated in duplicate and the experiment was done three times.

### Protein extracts and western blot analysis of autophagy markers

OE33 and OACM5.1C cells were incubated for 8 and 24 h with esomeprazole (0–200 μM). For the study of the basal autophagic flux, cells were cultured for 24 h with esomeprazole in the presence or absence of 10 μg/mL of the lysosomal inhibitors E-64d and pepstatin A (Sigma-Aldrich). For the experiments of autophagy induction, cells were cultured with esomeprazole in complete RPMI-1640 media or HBSS for 24 h. Whole-cell protein extracts were obtained by lysing cell pellets in lysis buffer supplemented with protease inhibitors and protein content was quantified employing the “BCA Protein Assay Kit” (Pierce, Thermo Scientific). SDS-PAGE was performed on acrylamide gels (8–15%) loading 50 μg of proteins per lane. Proteins were transferred to nitrocellulose membranes and incubated in TBS-T with 5% non-fat dry milk. Membranes were then incubated overnight at 4°C with primary antibodies: 1:1000 anti-LC3 (Sigma) and 1:1000 anti-p62 (Santa Cruz), and peroxidase-labeled secondary antibodies: anti-rabbit IgG from Vector Laboratories (Burlingame, CA, USA) at 1:5000 and anti-mouse antibody from Dako (Glostrup, Denmark) at 1:2000. Immunolabeling was detected using SuperSignal WestFemto (Pierce, Thermo Scientific, Waltham, USA), and visualized with a luminescent image analyzer (FUJIFILM LAS 3000, Fujifilm, Barcelona, Spain). MultiGauge software version 3.0 was used for densitometric analysis.

### RNA extraction and qPCR analysis of p62

Real time RT-PCR was performed using mRNA of cell cultures treated with vehicle or esomeprazole (1–200 μM) for 8 and 24 h. Total RNA from EAC cell lines was isolated using RNeasy Mini Kit (Qiagen, Madrid, Spain) and quantified (NanoDrop ND-1000, Wilmington, USA). Two micrograms of total RNA was reverse-transcribed with the Prime Script RT reagent Kit (Takara, Otsu, Japan). Real-time PCR was performed with the Prime Script Reagent Kit Perfect Real Time (Takara) in a thermo cycler LightCycler (Roche Diagnostics). Specific oligonucleotides for human p62 (5′-wordggttgccttttccagtgacg-3′, 5′-tcgcagacgctacacaagtc-3′) and human β-actin (5′-wordggacttcgagcaagagatgg-3′, 5′-agcactgtgttggcgtacag-3′) expression were used as a housekeeping gene. The threshold cycle (CT) was determined, and relative gene expression was expressed as follows: change in expression (fold) = 2^−Δ(ΔCt)^ where ΔCt = Ct (target)−Ct (housekeeping), and Δ(ΔCt) = ΔCt (treated)−ΔCt (control).

### Statistical analyses

Data analysis was performed using SPSS Statistics (IBM, New York, USA) and Graphpad (GraphPad Software, La Jolla, USA). Differences between groups were analyzed by student *T*-test or by ANOVA as appropriate. Data are expressed as mean ± *SE*.

## Results

### V-ATPase is expressed along the carcinogenic sequence of BE and also in BE and EAC cell lines, which show differences in the location of the pump

**Figure 1:**
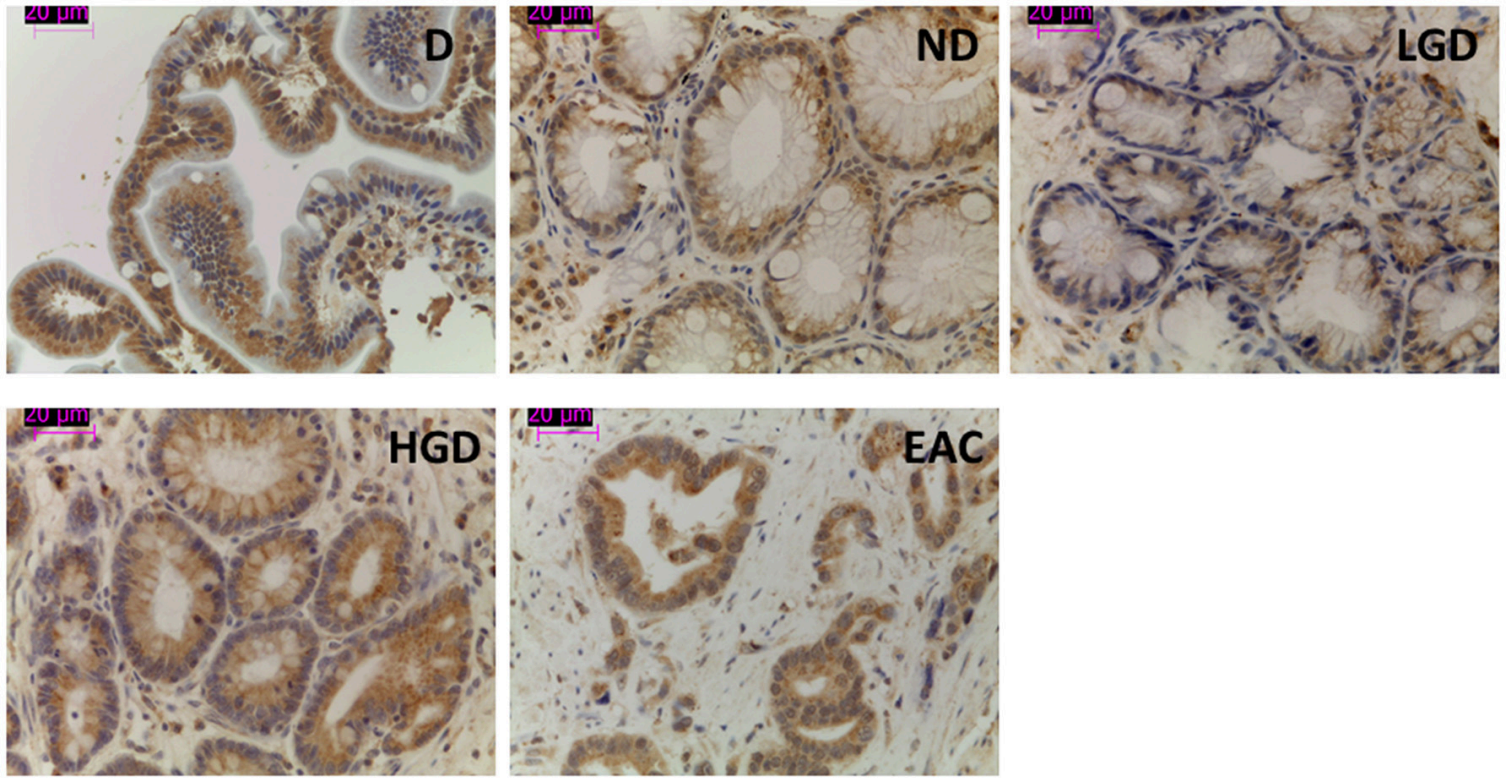
**Representative images of V-ATPase expression in BE and EAC in biopsy samples**. Immunohistochemical labeling of V-ATPase (brown staining) in paraffin-embedded biopsy samples corresponding to duodenum and the different stages of neoplastic progression in BE and EAC.

**Figure 2:**
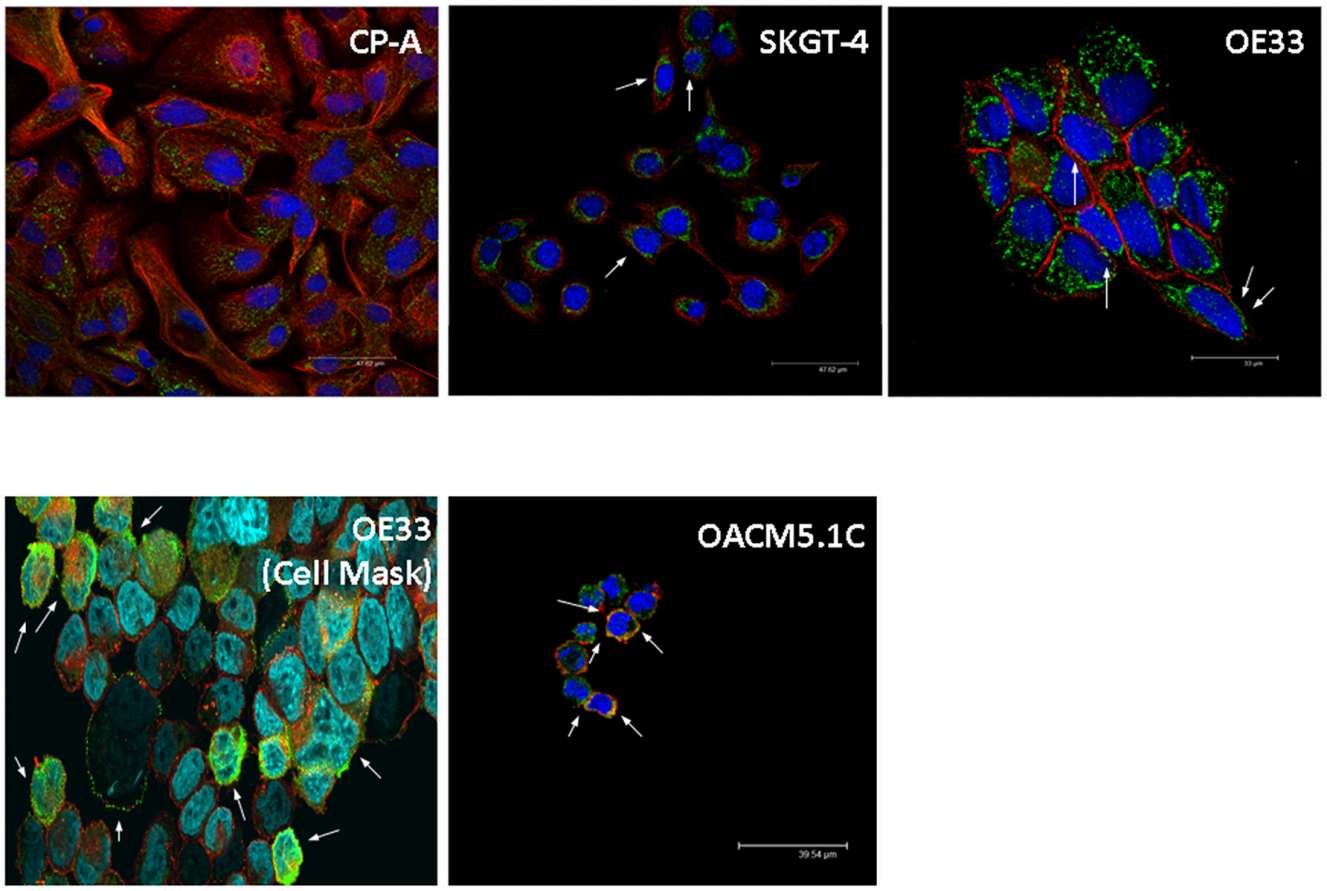
**Representative images of V-ATPase expression in BE and EAC cell lines**. Confocal microscopy showing V-ATPase (green dots) and plasma membrane (red/orange staining) in BE and EAC cell lines.

From an overall of 21 biopsies selected, 6 samples were diagnosed as ND, 4 LGD, 4 HGD, and EAC arising in BE was found in 7 specimens. The results showed that the pump is expressed in all the stages of neoplastic progression in BE (Figure 1). As shown in Figure 2, immunofluorescence revealed V-ATPase expression in all the cell lines evaluated. Barrett's cell line showed cytosolic expression of the pump, whereas the non-metastatic tumor cell lines OE33 and SKGT-4 showed similar staining patterns, exhibiting prominent cytosolic expression of V-ATPase with inconspicuous plasma membrane expression. To clarify whether OE33 cells expressed V-ATPase at plasma membrane or not, cells' edges were also labeled using Cell Mask stain. The results showed again a cellular subpopulation displaying expression of the pump at plasma membrane.

In contrast, the metastatic cells displayed cytosolic staining and also an apparent colocalization with phalloidin, suggesting that in these cells the pump is also located at the cell surface.

### Esomeprazole induced apoptosis and reduced cell proliferation of EAC cells

We first evaluated the proapoptotic effects of omeprazole and esomeprazole on EAC cells. Since we could observe that both PPIs significantly increased apoptosis (data not shown) but esomeprazole displayed a more intense proapoptotic effect than omeprazole, we decided to use esomeprazole in our experiments. Given that PPIs are prodrugs which require an acidic pH to be activated, we evaluated the effects of esomeprazole under physiological (pH 7.4) or acidified (pH 6.5) medium conditions.

**Figure 3:**
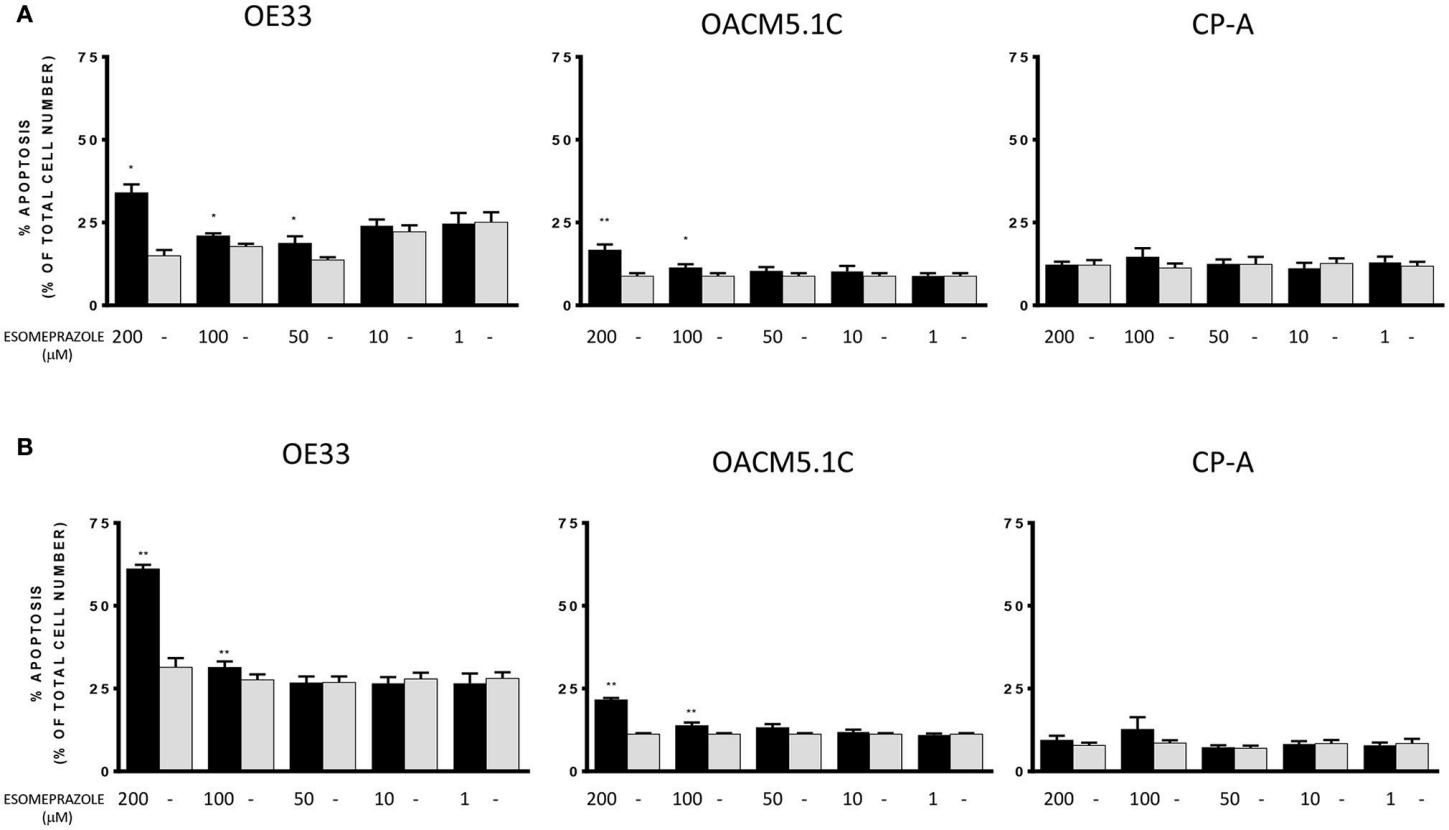
**Effects of esomeprazole on apoptosis of EAC and BE cell lines**. Apoptosis was evaluated in EAC and BE cell lines under physiological **(A)** or acidified **(B)** conditions. The bars represent the mean % of apoptosis in esomeprazole-treated cells with respect to control cells (DMSO only). All data are expressed as mean ± SEM of at least three independent experiments. Significant level ^*^p < 0.05; ^**^p < 0.01.

Esomeprazole significantly induced a dose-dependent increase in apoptosis of OE33 and OACM5.1C cancer cells in all the culture conditions evaluated, and had no effect on apoptosis of Barrett's esophagus cell line (Figure 3).

**Figure 4:**
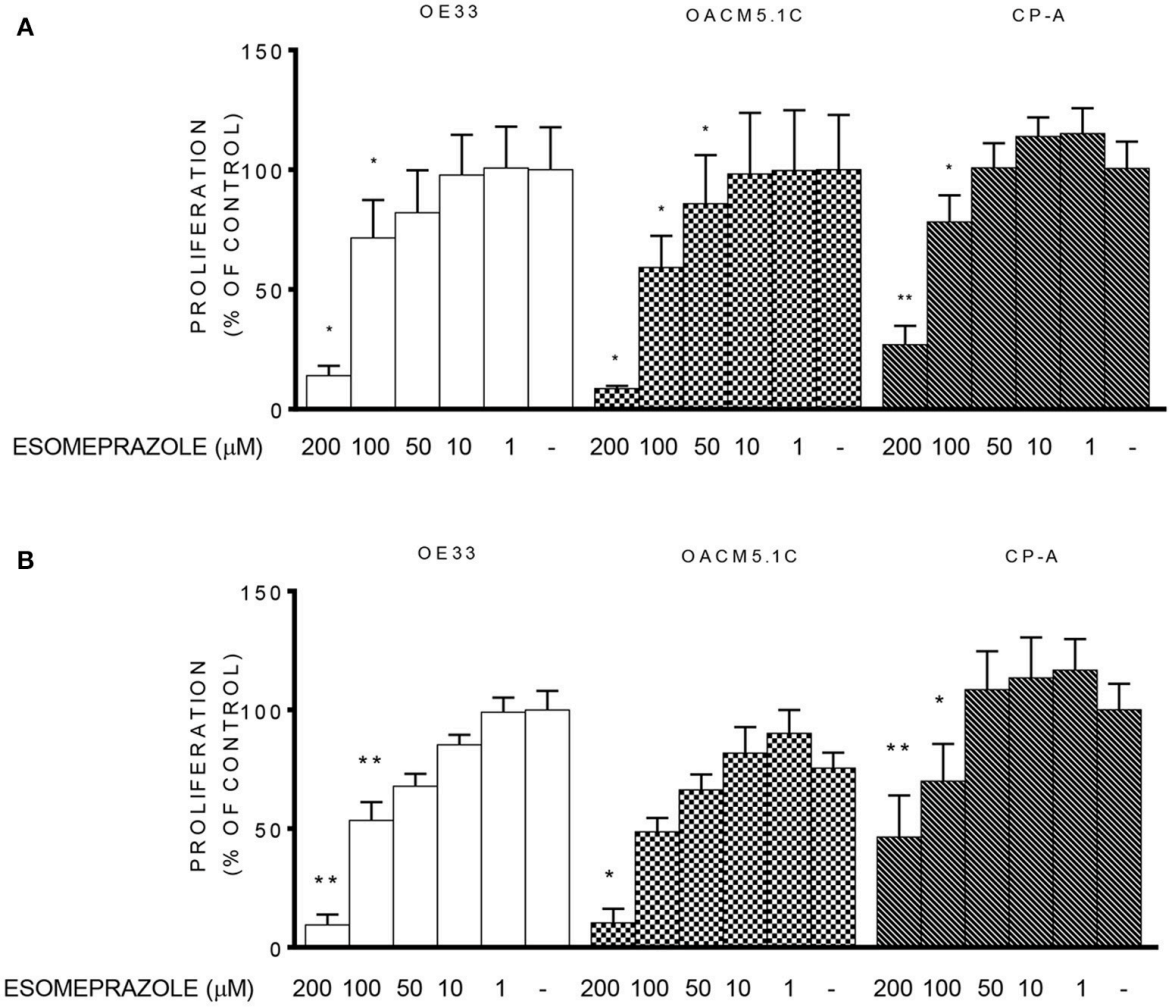
**Effects of esomeprazole on cell proliferation**. Cell proliferation in EAC and BE cell lines at physiological **(A)** or acidified **(B)** culture medium. The results are represented as the percentage of BrdU incorporation in esomeprazole-treated cells in comparison with untreated controls (DMSO only). All data are expressed as mean ± SEM of at least three independent experiments. Significant level ^*^p < 0.05; ^**^p < 0.01.

Since esomeprazole showed a clear induction of apoptosis of cancer cells without affecting the non-neoplastic cells, we sought to test the effects of PPI treatment on cell proliferation in EAC and in BE cell lines. The results observed show that treatment with esomeprazole significantly inhibited cell proliferation in the three cell lines evaluated both at physiological and acidic pH conditions (Figure 4).

### Esomeprazole diminished cell invasiveness of tumor cells

**Figure 5:**
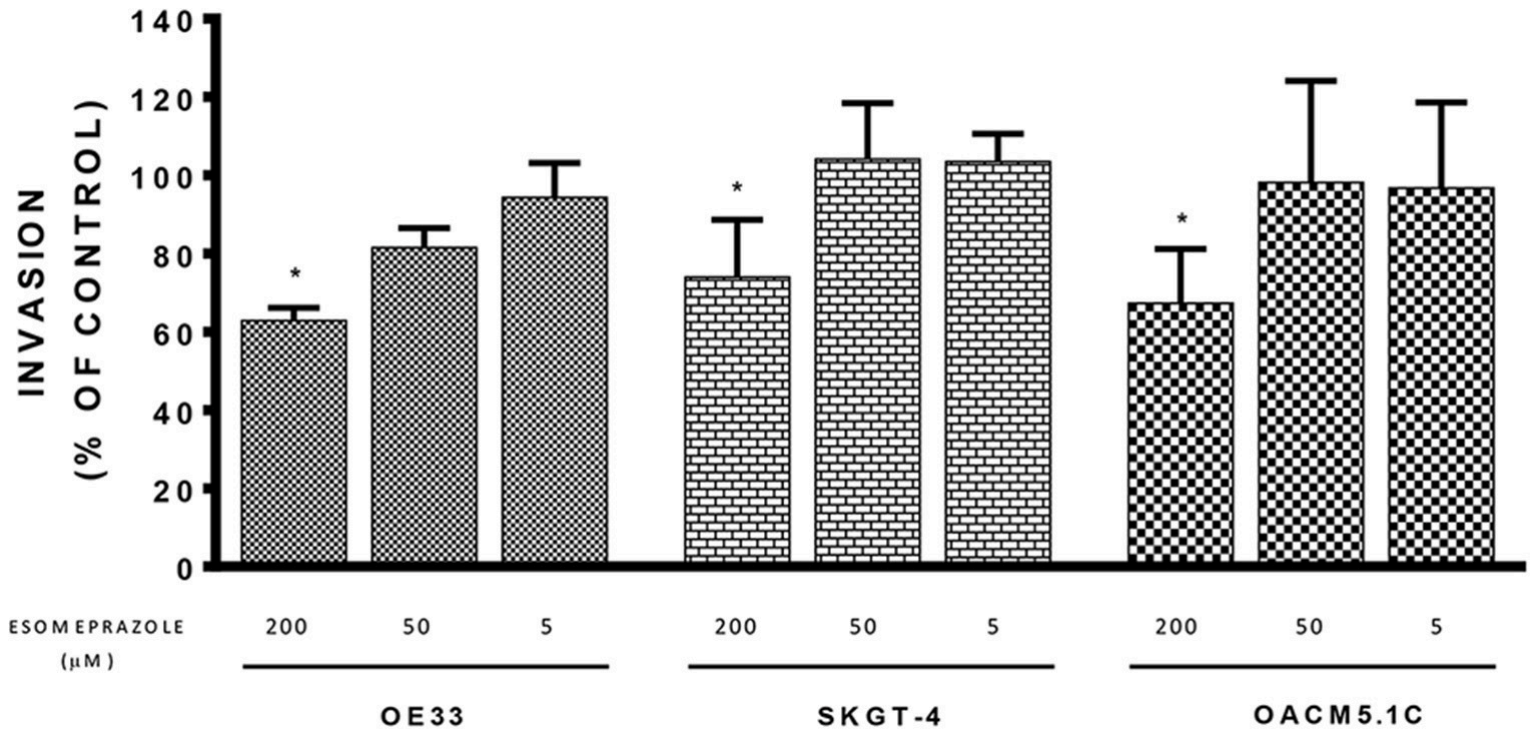
**Effects of esomeprazole on cell invasion**. The effects of esomeprazole on cell invasive properties of EAC cells were expressed as fluorescence values (RFUs) in esomeprazole-treated cells relative to untreated cells. All data are expressed as mean ± SEM of at least three independent experiments. Significant level ^*^p < 0.05.

We used a fluorometric assay that monitors the ability of tumor cells to migrate through the membrane basement layer to evaluate the effect that different concentrations of esomeprazole (5–200 μM) had on cell invasive properties. The results, expressed as % of fluorescence of cells treated with vehicle showed that the highest concentration of esomeprazole (200 μM) significantly reduced the invasive abilities of the three tumor cell lines (Figure 5): 62.87 ± 3.227% (*p* = 0.0206); 73.97 ± 5.529% (*p* = 0.0274) and 67.29 ± 5.244% (*p* = 0.0371) for OE33, SKGT-4 and OACM5.1C, respectively.

### Esomeprazole reduces intracellular pH of EAC but not BE cells

**Figure 6:**
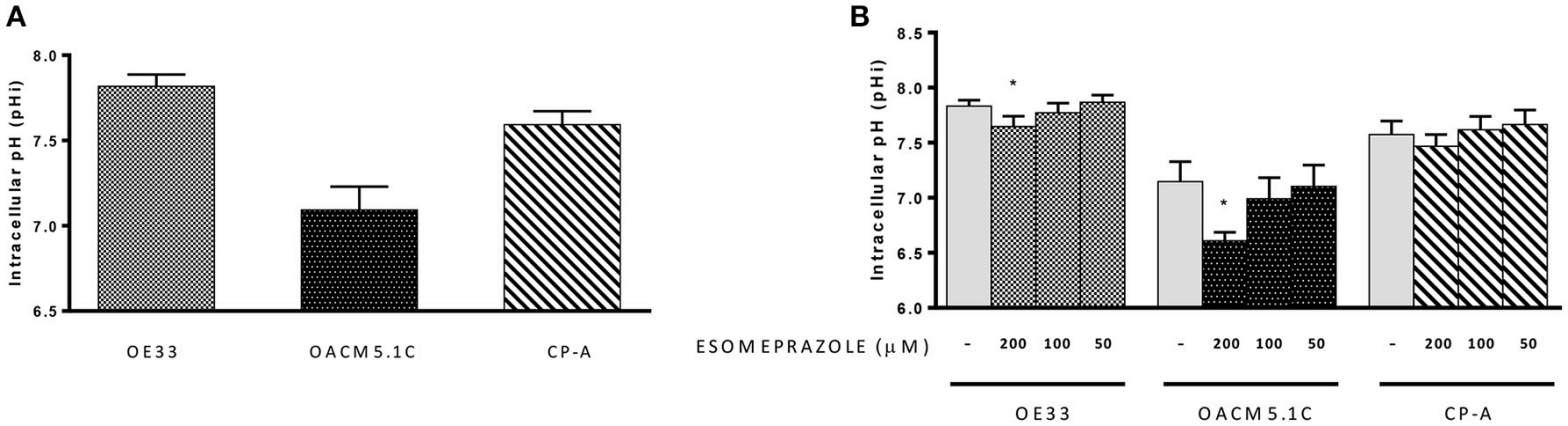
**Intracellular pH**. Basal pHi levels of OE33, OACM5.1C and CP-A cells **(A)**. Effects of esomeprazole on pHi **(B)**. Data are represented as the mean ± SEM of three independent experiments. Significant differences from the respective control values:^*^p < 0.05.

We evaluated whether treatment with esomeprazole (0–200 μM) for 24 h was able to induce changes in pHi in CP-A, OE33 and OACM5.1C cells. The analysis of basal pHi indicated that CP-A cells showed basal pHi-values of 7.59 ± 0.08, OE33 cells 7.82 ± 0.07 and metastatic cells OACM5.1C displayed more acidic basal pHi-values: 7.09 ± 0.135 (Figure 6A).

The highest concentration of esomeprazole caused a significant decrease of pHi in EAC cells OE33 (7.65 ± 0.09 vs. 7.82 ± 0.06 in control cells, *p* < 0.05) and OACM5.1C (6.61 ± 0.08 vs. 7.15 ± 0.18, *p* < 0.05) but had no effect on pHi of BE cells (Figure 6B).

### Esomeprazole-induced apoptosis is dependent on ROS production

**Figure 7:**
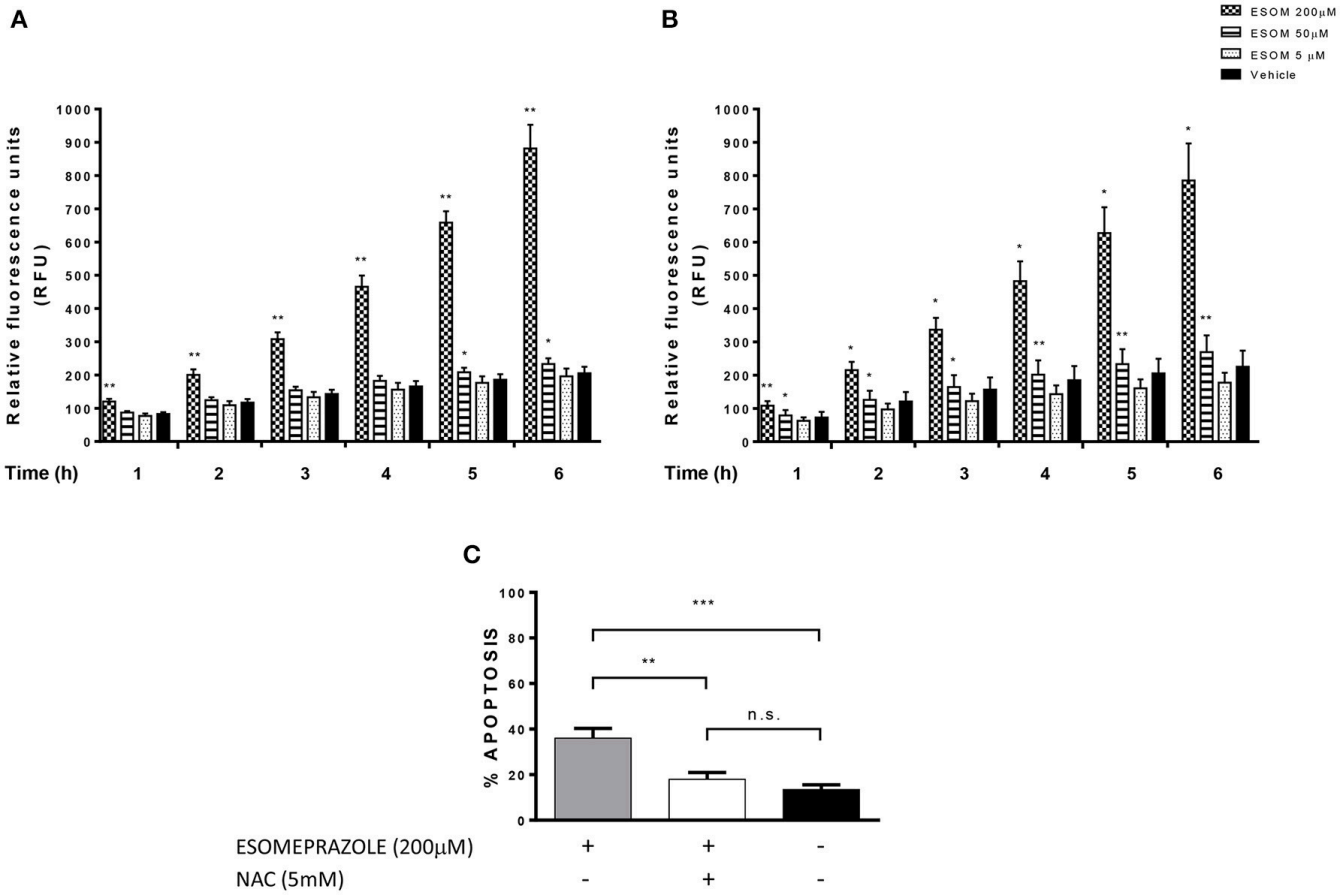
**Esomeprazole and ROS production in EAC cells**. ROS levels in esomeprazole-treated OE33 **(A)** and OACM5.1C **(B)** cells and effects of the antioxidant NAC on esomeprazole-induced apoptosis in OE33 cells **(C)**. All data are expressed as the mean ± *SEM* of at least three independent experiments. Significant differences: ^*^p < 0.05; ^**^p < 0.01; ^***^p < 0.001.

According to previously published data showing that PPI-induced cell death is mediated by ROS-dependent mechanisms in melanoma and lymphoma cells (De Milito et al., 2007; Marino et al., 2010), we evaluated the implication of ROS production in the effects triggered by esomeprazole in EAC cells. Firstly, we observed that the increase in ROS levels is an early event in EAC cells after the addition of the PPI, which occurs in a dose and time-dependent manner (Figures 7A,B). We also sought to assess the role of ROS in esomeprazole-induced apoptosis. In order to achieve this objective we first checked that preincubation with the ROS scavenger NAC diminished the levels of ROS in esomeprazole-treated cells (data not shown). After 48 h of treatment, esomeprazole induced apoptosis in OE33 cells and pretreatment with NAC significantly reduced esomeprazole-induced apoptosis to similar levels than control cells (Figure 7C).

### Esomeprazole diminished ΔΨ_m_ in OE33 but not in OACM5.1C cells and had no effect on cytochrome C release

Mitochondria plays a central role in the regulation of the apoptotic process. An opening of the mitochondrial permeability transition pore has been shown to induce depolarization of the mitochondrial membrane potential (ΔΨ_m_) and the release of pro-apoptotic proteins as cytochrome C (Hengartner, 2000; Ly et al., 2003). Due that esomeprazole increased ROS production in EAC cells and mitochondria is one of the main intracellular targets for oxidative stress we studied whether esomeprazole affected ΔΨ_m_ and the release of cytochrome C in EAC cells.

**Figure 8:**
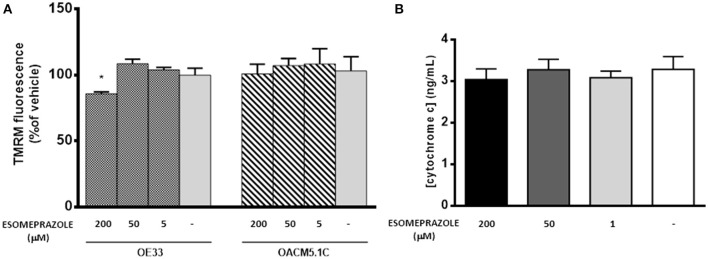
**Effects of esomeprazole on mitochondrial membrane potential (A)** and cytochrome C release from mitochondria to cytosol **(B)**. Data showed in **(A)** are represented as % of TMRM fluorescence intensity in esomeprazole-treated cells with respect to vehicle. Data showed in **(B)** are expressed as mean ± SEM of cytosolic concentration of cytochrome c in esomeprazole-treated OE33 cells. All experiments were repeated at least three times. Significant differences from the respective control values: ^*^p < 0.05.

As shown in Figure 8A, the highest concentration of esomeprazole significantly diminished ΔΨ_m_ in OE33 cells whereas it did not affect ΔΨ_m_ in the metastatic cell line OACM5.1C. Since depolarization of the ΔΨ_m_ seems to be a trigger for the release of proapoptotic proteins from mitochondria, we therefore evaluated the levels of cytosolic cytochrome c in OE33 cells. The results shown in Figure 8B revealed that esomeprazole did not induce the release of cytochrome c from mitochondria.

### Esomeprazole induces changes in autophagic activity in EAC cells

**Figure 9:**
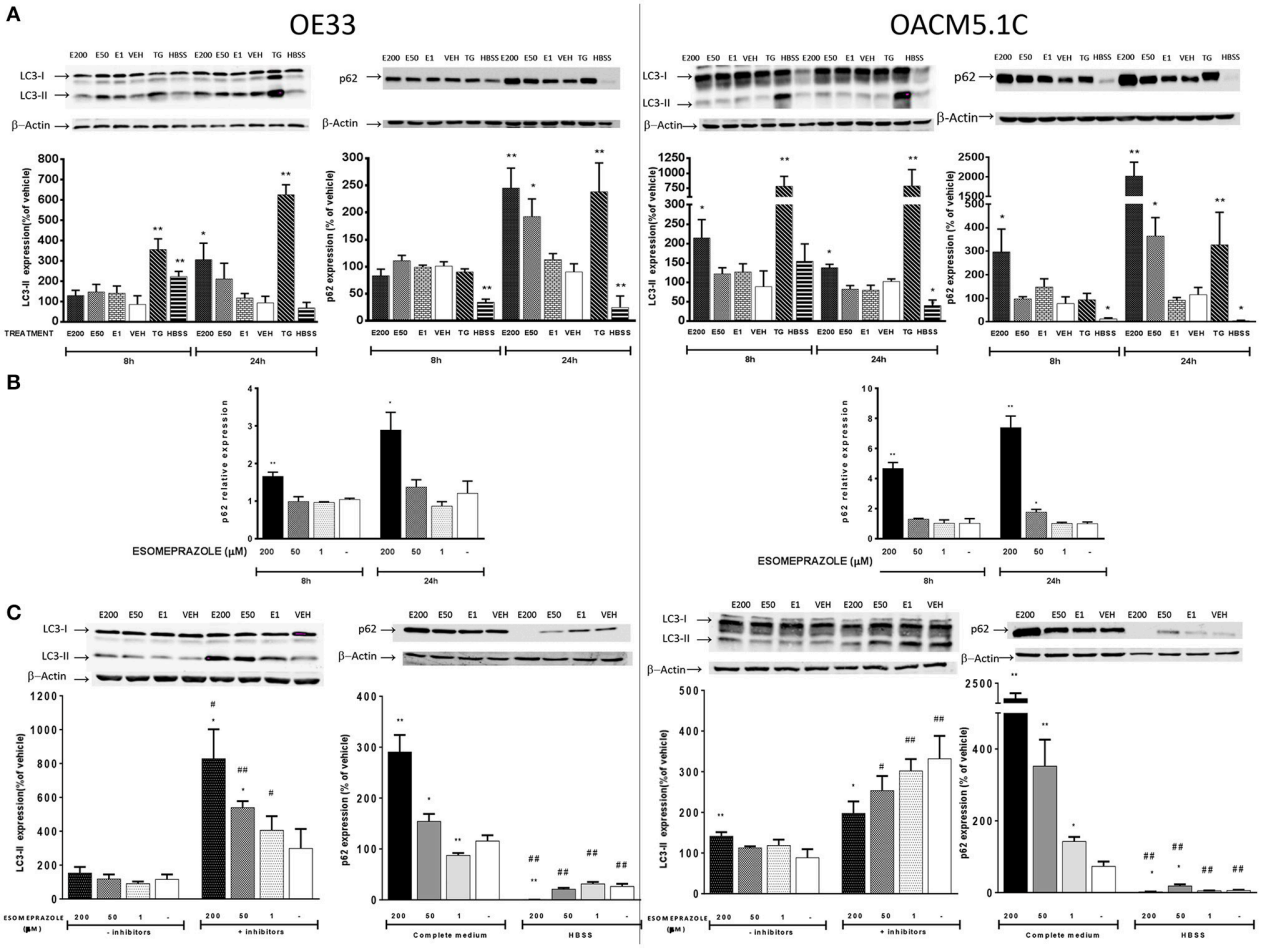
**Effects on autophagy of EAC cells**. OE33 and OACM5.1C cells were used. Autophagy markers LC3 and p62 were evaluated by WB after 8 and 24 h incubation with esomeprazole **(A)**. p62 relative expression **(B)** was evaluated by RT-PCR after treatment with esomeprazole for 8 and 24 h and the results are expressed as the level of expression of p62 with respect to cells treated with vehicle alone. β-actin was used as a housekeeper control. The effects of esomeprazole on autophagic flux **(C)** was evaluated by WB after incubating cells in complete media in the presence or absence of lysosomal inhibitors pepstatin A and E-64d, and in complete media or HBSS for 24 h. WB representative gels are lined up their respective results and all data are represented as the % of expression of autophagy markers LC3-II or p62 with respect to values obtained in cells treated with vehicle alone. All data are represented as mean ± SEM of three independent experiments. Significant differences from the respective control values: ^*^p < 0.05; ^**^p < 0.01. Significant differences from the respective treatment in complete medium or medium without inhibitors: ^#^*p* < 0.05; ^##^*p* < 0.01.

We evaluated by western blot (WB) the levels of the autophagy markers LC3 and p62 in OE33 and OACM5.1C cells after 8 and 24 h of ESOM treatment. LC3 is detected as two bands following SDS-PAGE: LC3-I and LC3-II and in conditions of autophagy activation the amount of LC3-II increases which is frequently used as an autophagy activation indicator (Mizushima and Yoshimori, 2007). p62 is used as an indicator of autophagic flux whose levels decrease when there is an active autophagic flux within the cell (Moscat and Diaz-Meco, 2009). The results showed in Figure 9A revealed that, in OE33 cells, esomeprazole did not produce changes in neither LC3-II nor p62 levels after 8 h of treatment. However, after 24 h of treatment esomeprazole increased the levels of both proteins: LC3-II at 200 μM and p62 at 200 and 50 μM, respectively. In the metastatic cell line OACM5.1C, esomeprazole led to an increase in the expression of both proteins after 8 and 24 h of treatment (Figure 9A).

Increased LC3-II levels may be explained by induction of autophagic activity or due to accumulation of autophagosomes as a consequence of impaired degradation of autolysosome content. In addition, accumulation of p62 may be an indicator of a blockade in the autophagic flux. However, as shown previously, p62 levels might also be raised as a consequence of increased p62 transcription induced by ROS (Mathew et al., 2009; Jain et al., 2010). To clarify whether p62 increased levels were a consequence of its increased expression, we evaluated the effects of esomeprazole on p62 gene expression by quantitative RT-PCR. The results showed that esomeprazole induced an increase in p62 expression in both cell lines after 8 and 24 h (Figure 9B) which suggests that the increase in p62 levels observed might be due to a rise in mRNA expression rather than as a result of a blockade of autophagolysosome degradation. To further elucidate this question, we evaluated basal autophagic flux by assessing the levels of the autophagic marker LC3-II in the presence or absence of lysosomal protease inhibitors E-64d and pepstatin A. An increase in LC3-II levels in the presence of lysosomal inhibitors is considered an evidence of an efficient autophagic flux, while a decrease would indicate a failure in the autophagic process which happened before lysosomal degradation (Barth et al., 2010). The results showed that esomeprazole effects on autophagic flux are opposite in the two tumor cell lines. Esomeprazole (200 and 50 μM) significantly increased LC3-II levels in OE33 cells, but the PPI decreased the levels of the autophagic marker in the metastatic cell line at the highest concentration (Figure 9C), which indicates that the PPI blocks basal autophagic flux in OACM5.1C cells at some point before lysosomal degradation.

Another experimental approach to study basal autophagic flux consists in evaluating the levels of an autophagy marker after the induction of autophagy and the subsequent addition of the treatment with the substance of interest. We induced autophagy by nutrient deprivation which is achieved by incubation of the cells in HBSS instead of complete culture medium. The results showed that in both EAC cell lines, the levels of the autophagic marker p62 were significantly lower in cells incubated in HBSS, indicating an effective activation of autophagy (Figure 9C). OE33 and OACM5.1C cells treated with the highest concentration of esomeprazole displayed a prominent decrease in p62 levels in comparison to cells treated with vehicle alone, indicating that in both non-metastatic and metastatic EAC cells esomeprazole did not produce a blockade in induced autophagy (Figure 9C).

## Discussion

V-ATPase plays a key role in tumor pH homeostasis and PPIs have shown to inhibit this pump (Mattsson et al., 1991; Mizunashi et al., 1993; Moriyama et al., 1993), thus exerting antineoplastic effects in different tumors (Marquardt and Center, 1991; Palanca-Wessels et al., 1998; Martínez-Zaguilán et al., 1999; Luciani et al., 2004; De Milito et al., 2007; Udelnow et al., 2011; Ishiguro et al., 2012).

It has also been reported that the degree of expression of this pump increases in parallel with neoplastic transformation in other tumors (Lu et al., 2005). This is, to our knowledge, the first study to date evaluating V-ATPase expression along the Barrett's carcinogenetic sequence, and the results showed the presence of the pump in all BE and EAC glands studied, which suggests the possibility of evaluating this factor as a future therapeutic target in the treatment of EAC.

To date, several pre-clinical studies have shown that the administration of PPIs may exert direct cytotoxic effects on tumor cells by disturbing the H^+^ transport dynamics and also may increase the efficacy of anticancer drugs, restoring chemotherapeutic sensitivity in drug-resistant cancer cells (Marquardt and Center, 1991; Palanca-Wessels et al., 1998; Martínez-Zaguilán et al., 1999; Luciani et al., 2004; De Milito et al., 2007; Udelnow et al., 2011; Ishiguro et al., 2012; Azzarito et al., 2015; Lugini et al., 2016).

Due the positive results of these studies, we sought of great interest to evaluate whether PPIs also exerts *in vitro* antitumor effects on EAC cells and the cellular mechanisms underlying these effects, which have not been deeply studied yet. In our study we decided to evaluate the antineoplastic effects of omeprazole and esomeprazole, two of the more widely used PPIs in the treatment of acid-related diseases. Since esomeprazole is a more potent gastric acid secretion inhibitor than omeprazole and because we observed that esomeprazole was more effective inducing apoptosis in our model that omeprazole (data not shown), we decided to use esomeprazole in our experiments. First, we assessed if V-ATPase was present in the cell lines used in our study, which was a requirement for the use of these cells in our study; and we later evaluated whether the expression was at plasma membrane level or not. Confocal microscopy results showed that V-ATPase was expressed in all the cell lines studied, with most of the subcellular populations displaying cytosolic staining in BE and non-metastatic EAC cell lines. However, the expression at the plasma membrane seemed to be increased in the metastatic cell line OACM5.1C, which is in accordance with previous data obtained in other tumors (Martinez-Zaguilan et al., 1993).

Proton pump inhibitors are administered as inactive prodrugs which need low pH to convert in to the active form. Previous studies have shown that cancer cells can overcome an acidification of the culture media (Yeo et al., 2004), so we thought of interest to evaluate whether the *in vitro* effects of PPI on cancer cells would be potentiated by low pH culture medium, conditions. We decided to evaluate apoptosis after 48 h of treatment at physiological pH and after 24 h at acidic pH based on previous studies with our cell model in which we observed only a slight increase in apoptosis when apoptotic inducers were used for <48 h at physiological pH. However, after 48 h of esomeprazole treatment at acidic pH, most of the cultures died, indicating that cells could not overcome the adverse conditions induced by esomeprazole at low pH. These findings point to higher cytotoxic effects of PPI with lower pH values, as is the case of tumor surrounding microenvironment, suggesting the potential clinical applications of PPIs as anticancer agents.

Our results showed that esomeprazole, especially at high concentrations (50, 100, and 200 μM) increased apoptosis of EAC cells without affecting BE cells, which suggests that this effect may be exclusive of tumor cells. As seen in other studies of the antineoplastic action of PPIs, the proapoptotic effects were observed only when the highest concentration of the drug was used (Marquardt and Center, 1991; Palanca-Wessels et al., 1998; Luciani et al., 2004; Yeo et al., 2008; Udelnow et al., 2011), far from the levels achieved during normal acid suppression in patients with BE. During normal acid suppression (20 mg/day), the maximum plasma levels achieved are about 7 μM (Katagiri et al., 2005). However, higher plasma levels can be achieved in special dosage schedules as is the case of Zollinger-Ellison syndrome (120–240 mg/day; Frucht et al., 1991). In addition, the drug delivery route can also modify plasma levels of esomeprazole. Thus, intravenous administration could increase plasma levels of esomeprazole compared to oral intake (Junghard et al., 2002). On the other hand, individual plasma levels achieved after PPI administration should be considered given that they are extremely variable between subjects. Omeprazole, esomeprazole, and lansoprazole are extensively metabolized by CYP2C19 and CYP3A4, and CYP2C19 polymorphisms can significantly influence the metabolism of PPIs. As a consequence, some patients could achieve higher levels of the PPI based on their genotype as higher levels are found in slow metabolizers (Furuta et al., 2002). In addition, because of the trend of PPIs to accumulate in tissues displaying low pH-values, it may be possible to achieve high concentrations of the drug at acidic compartments—as the tumor site—with the administration of relatively low doses of PPI. In this context, it would be interesting to study the tissue concentrations of esomeprazole observed after the PPI intake to further establish the optimum dosage necessary in cancer treatment.

The results of the proliferation assays showed that esomeprazole addition at high concentrations diminished cell proliferation, which also agrees with the effects of PPIs observed in other tumors (Marquardt and Center, 1991; Palanca-Wessels et al., 1998; Luciani et al., 2004; Yeo et al., 2008; Udelnow et al., 2011). However, we observed that the highest concentrations of esomeprazole also inhibited proliferation in BE cells too. Unlike most of the studies performed to date, we included a non-tumor cell line which corresponds to the precursor lesion of the tumor, as a control to assess if the effects of the treatments evaluated were exclusive of EAC cells or not. Barrett's mucosa has higher proliferative rate than healthy squamous esophagus (Mathew et al., 2009), and indeed a reduction in the proliferative rate in BE cells could represent a benefit in terms of neoplastic progression because it can reduce the mutation rate and the risk for neoplastic progression. However, the antiproliferative effects observed were produced only at 100 and 200 μM, and no effect was observed at lower concentrations, which are the circulating levels achieved during a standard acid-suppressive therapy with PPIs.

The highest concentration of PPI evaluated also showed to inhibit the invasive properties of EAC cells in a significant manner. Taken together, the results obtained indicate that PPIs at high concentrations exert *in vitro* antineoplastic effects on EAC cells, which suggests the interest to extend the studies on this tumor elucidating the cellular mechanisms involved in these effects. To this respect the few studies existing to date focused on the mechanisms underlying the antineoplastic effects of PPIs indicate that they may depend on the cell line evaluated.

One of the mechanisms associated with the antitumor effects of PPIs previously reported (Martínez-Zaguilán et al., 1999; Matsuyama et al., 2000) is the reversal of the abnormal pH gradient existing in cancer cells. The results obtained in our experiments reflect, on the one hand, the differences existing between the different cell lines used in this study. Non-metastatic tumor cells displayed a basal pHi slightly higher than normal cells, which is in accordance to previous reports (Ouatu-Lascar and Triadafilopoulos, 1998; Ouatu-Lascar et al., 1999) but the metastatic cell line showed surprisingly an acidic pHi. A comparison between pHi of tumor cells with distinct invasive properties has not been studied yet, but due the potential implications of pHi in tumor progression it might be interesting to study this fact in greater depth.

On the other hand, our results showed that esomeprazole had no significant effects on pHi of BE cells but, as previously seen in other tumors (De Milito et al., 2007, 2010) the use of high concentrations of esomeprazole significantly lowered pHi of cancer cells. Studies in melanoma, gastric cancer, and B-cell lymphoma showed that PPIs induced an important decrease of pHi (about 0.5 pH units or higher), thus creating the optimal conditions for the activation of caspases and different apoptotic pathways. In our cell model esomeprazole diminished pHi of tumor cells, but we could observe an important decrease only in the metastatic cell line OACM5.1C. In contrast, OE33 cells only showed a slight decrease in intracellular pH upon the addition of esomeprazole, suggesting that the alteration in pHi might not be a key mechanism driving the antineoplastic effects of PPIs in this cell line and reveals again the differences existing between different EAC cell lines.

An increase in ROS production has also been related to the antineoplastic effects of PPIs (von Schwarzenberg et al., 2013), and previous reports showed that PPIs increased ROS production in melanoma and lymphoma cell lines (Marquardt and Center, 1991; Palanca-Wessels et al., 1998). In our model, esomeprazole also increased ROS levels in a time and concentration-dependent manner and ROS seemed to play a key role in the cytotoxic effects of esomeprazole on EAC cells, since the addition of the ROS scavenger NAC completely abolished the proapoptotic effects of esomeprazole. An increase in ROS levels is related to oxidative stress, and damages mitochondrial membrane thus altering its Ψ_m_ and allowing the release of proapoptotic molecules to the cytosol (Cregan et al., 2004). In our model, the highest concentration of esomeprazole evaluated decreased ΔΨ_m_ in OE33 cells while had no effect on metastatic cells OACM5.1C, which shows the differences existing between the two EAC cell lines. Unlike previous reports in melanoma and lymphoma cells (Marquardt and Center, 1991; Palanca-Wessels et al., 1998), in EAC cells esomeprazole did not induce the release of cytochrome C from the mitochondria, which suggests that in our model PPI-induced apoptosis might be independent from the caspase activation pathway.

One of the consequences of high levels of ROS is autophagy induction (Scherz-Shouval et al., 2007). Autophagy is an adaptative mechanism which contributes to cancer cells survival in unfavorable conditions as hypoxia, nutrient starvation, or cytotoxic agents (Palanca-Wessels et al., 1998; Martínez-Zaguilán et al., 1999). The role of autophagy in cancer is controversial since it seems that it could decrease tumorigenesis but also help cancer cells to overcome the toxicity induced by the antineoplastic agents (Palanca-Wessels et al., 1998; Martínez-Zaguilán et al., 1999; Mizushima et al., 2008; White et al., 2010). We evaluated the effects of esomeprazole in the autophagic markers LC3 and p62 in EAC cells, and the results showed that esomeprazole increased both two markers, which may be an indicator of an activation of autophagy and further blockade of autophagic flux. However, quantitative RT-PCR revealed that esomeprazole also increased the expression of p62 in OE33 and OACM5.1C cells, indicating that the increase in p62 levels seen in WB might be a consequence of activation at the transcription level rather than a blockade in autophagic flux. To further elucidate the effects of esomeprazole on autophagic flux we evaluated basal and induced autophagy, and the results showed that the effects differ upon the cell line studied. In the metastatic cells, the results suggest that esomeprazole blocks basal autophagy and this blockade happens at some point before autolysosome degradation. These results agree with the activation and further blockade of autophagic flux observed previously in pancreatic cancer and melanoma (De Milito et al., 2007; Udelnow et al., 2011), in whose autophagy seemed to act as a protective mechanism against the damage induced by esomeprazole. Of note, esomeprazole did not alter basal autophagy in non-metastatic cells, which could be a sign of differences in the mechanisms involved on the cytotoxic effects of PPIs on EAC cells. On the other hand, once autophagy was induced by HBSS, esomeprazole diminished p62 levels with respect to control cells in both EAC cell lines, indicating that the PPI did not block induced autophagic flux neither in OE33 nor in OACM5.1C cells. The results of the evaluation of autophagy are very interesting and reveal again the differences existing between the two EAC cells evaluated. While the activation of autophagy and further blockade of autophagic flux induced by PPI shown in the metastatic cells agree with previous reports observed in melanoma and pancreatic adenocarcinoma (De Milito et al., 2007; Udelnow et al., 2011), esomeprazole did not block basal autophagy in the non-metastatic cell line, suggesting that the role of autophagy in the cytotoxicity induced by PPIs may be different depending on the cell line evaluated.

In conclusion, the present study demonstrates that PPI exerts antineoplastic effects on EAC *in vitro*. PPIs induced a decrease in pHi of EAC cells and an increase in ROS levels, which is responsible of the proapoptotic effects observed. However, the cellular mechanisms involved in those effects seem to vary upon the cell line evaluated. Our study suggests that mechanisms involved in ROS production and regulation of pHi may be relevant targets for new antitumor strategies and points to the possibility to use PPIs as antineoplastic drugs against esophageal adenocarcinoma.

## Author contributions

EC performed most experiments and wrote the paper. NA was involved in the design of the study and the edition of the manuscript. JE provided vital reagents and analytical tools and contributed to the edition of the manuscript. MG was involved in cell culture studies and immunohistochemical analysis. AL was involved in the revision of the work and provided analytical tools. EP was involved in the conception and design of the study, provided vital reagents and analytical tools, and collaborated in the edition of the paper.

## Funding

This study was supported with a grant from Instituto de Salud Carlos III (PI14/01931) and Sociedad Aragonesa de Patología Digestiva.

### Conflict of interest statement

The authors declare that the research was conducted in the absence of any commercial or financial relationships that could be construed as a potential conflict of interest.
